# Effects of Standardized Natural Citrus Extract on Growth, Gut Health, Carcass Quality, and Welfare of Broiler Chickens

**DOI:** 10.3390/ani15020127

**Published:** 2025-01-08

**Authors:** Sekhou Cisse, Arkadiusz Matuszewski, Damian Bień, Patrycja Ciborowska, Anna Zalewska, Jakub Urban, Monika Michalczuk, Marta Mendel, Urszula Latek, Joanna Polak, Małgorzata Sobczak-Filipiak, Paweł Konieczka, Mohammed El Amine Benarbia

**Affiliations:** 1 Joint Lab ANR FeedInTech (FIT: SONAS/Nor-Feed), 49070 Beaucouzé, France; amine.benarbia@norfeed.net; 2 Nor-Feed SAS, 3 rue Amédéo Avogadro, 49070 Beaucouzé, France; 3 Department of Animal Environment Biology, Institute of Animal Sciences, Warsaw University of Life Sciences (SGGW), Ciszewskiego 8, 02-786 Warsaw, Poland; arkadiusz_matuszewski@sggw.edu.pl; 4 Division of Animal Nutrition, Institute of Animal Sciences, Warsaw University of Life Sciences, Ciszewskiego 8, 02-786 Warsaw, Poland; damian_bien@sggw.edu.pl; 5 Department of Animal Breeding, Institute of Biology, Warsaw University of Life Sciences, Ciszewskiego 8, 02-786 Warsaw, Poland; patrycja_ciborowska@sggw.edu.pl (P.C.); anna_zalewska1@sggw.edu.pl (A.Z.); jakub_urban@sggw.edu.pl (J.U.); monika_michalczuk@sggw.edu.pl (M.M.); 6 Department of Preclinical Sciences, Division of Pharmacology and Toxicology, Institute of Veterinary Medicine, Ciszewskiego 8, 02-786 Warsaw, Poland; marta_mendel@sggw.edu.pl (M.M.); urszula_latek@sggw.edu.pl (U.L.); joanna_polak@sggw.edu.pl (J.P.); 7 Department of Pathology and Veterinary Diagnostics, Institute of Veterinary Medicine, Warsaw University of Life Sciences, Nowoursynowska 159C, 02-776 Warsaw, Poland; malgorzata_sobczak_filipiak@sggw.edu.pl; 8 Kielanowski Institute of Animal Physiology and Nutrition Polish Academy of Sciences, Instytucka 3, 05-110 Jabłonna, Poland; pawel.konieczka@uwm.edu.pl; 9 Department of Poultry Science and Apiculture, University of Warmia and Mazury in Olsztyn, Oczapowskiego 5, 10-719 Olsztyn, Poland

**Keywords:** citrus extract, growth performance, carcass quality, gut health, welfare

## Abstract

This study aimed to investigate the effects of a Standardized Natural Citrus Extract (SNCE) on broiler chickens’ growth performance, gut health, carcass quality, and welfare. A total of 756 one-day-old Ross 308 males were randomly assigned to two groups: a control group (CTL) fed with a standard diet, and a citrus group (SNCE) fed with the same standard diet supplemented with 250 g/ton of feed of SNCE. Growth performance was recorded weekly until d 35, while mortality was recorded daily. The feed conversion ratio (FCR) and European Efficiency Index (EEI) of broiler chickens were also calculated weekly. At day 35, 10 birds per group were randomly selected for slaughter performance. In parallel, broiler chickens’ welfare was assessed according to the Welfare Quality Assessment Protocol. Caecal digest was also collected post mortem for short-chain fatty acids (SCFA) analyses, and jejunum samples were collected for ex vivo gut permeability assay. SNCE dietary supplementation enhanced broiler chickens’ performance, i.e., final bodyweight and EEI, compared to the CTL group. The carcass weight was also significantly higher in the SNCE group. In addition, the fat percentage was lower in the SNCE group. Regarding broiler chickens’ welfare and gut health parameters, Footpad Dermatitis (FPD) and gate score were also lower in birds supplemented with SNCE. The SCFA measurement showed a lower concentration of iso-butyric acid, iso-valeric acid, and total putrefactive SCFA in the SNCE group. The differences in gut permeability measured as TEER value indicate that using citrus extract lowered the risk of gut inflammation. This study provides valuable insights into the mechanisms of action that may underlie the observed effects of SNCE on performance, as demonstrated in this study and others. These effects could potentially be attributed to the reduction in inflammation and the enhanced utilization of nutrients. Further studies are needed to confirm these results.

## Simple Summary

Improving animal growth performance while maintaining good health and welfare is a serious challenge that poultry production is facing. Using feed additives could be part of the solution. Here, we evaluate the effect of a Standardized Natural Citrus Extract (SNCE) on growth performance, animal welfare, and the intestinal health of broiler chickens. The main results showed that SNCE supplementation in feed improved the final bodyweight, the European Efficiency Index, and the carcass quality of broiler chickens. Regarding welfare, the occurrence of Footpad Dermatitis was reduced in birds supplemented with SNCE. Gut health was also improved, as seen by the measurable indicators such as putrefactive short-chain fatty acids and gut integrity in birds that received the SNCE. Based on these results, we suggest the use of SNCE as feed additive to improve growth performance, health, and welfare of broiler chickens.

## 1. Introduction

For decades, the livestock production sector has focused on improving animal production efficiency to stretch out the world population’s needs concerning farmed products [1]. This is particularly true for conventional broiler chickens’ production, where advancements in genetics, nutrition programs, management strategy, and medicine have resulted in more efficient birds, allowing a decrease of the slaughter age dramatically [2,3]. This is well illustrated by the fact that the broiler chickens’ live weight at 42 days of age increased by over 300% in the last 50 years [4,5]. However, this high productive capacity level of broiler chickens raised concerns about animal welfare [4,6] and meat quality [2,3]. Indeed, the incidence of disorders such as skin lesions and lameness has increased in recent years [4,7]. These issues generate important economic losses in the broiler chicken production sectors. The current challenges of broiler chicken production consist of maintaining good productivity and taking into account the societal demands regarding the production systems [8]. Indeed, animal welfare concerns as well as livestock production system sustainability have become more and more important for consumers in the last two decades in many regions of the world [8,9]. This is the reason why there is a need for more renewable development strategies to maintain broiler chickens’ efficiency while improving poultry welfare and meat quality.

Interestingly, studies have shown that maintaining intestinal health is closely linked to both productivity and well-being [10]. That is why maintaining intestinal health is crucial to ensure the good productivity and welfare of broiler chickens. Indeed, good intestinal health is essential for nutrient digestion and absorption, leading to better animal performance [10]. Moreover, gut health issues are often associated with a decline in welfare parameters related to skin integrity [11]. Intestinal health can be managed in several ways. Among existing solutions, supplementing broiler chickens with feed additives is a good one [12,13,14]. These solutions include, among others, plant and plant extracts, organic acids, probiotics, prebiotics, and exogenous enzymes. In most cases, these solutions would play a central role in gut health, resulting in increased gut integrity, well-balanced microbiota, and nutrient digestion and absorption, as well as reduced ammonia and moisture content in their excreta, which may indirectly improve welfare parameters linked to skin integrity [15,16].

Citrus extracts have been widely used in animal nutrition as feed additives to improve feed efficiency due to the beneficial effect they confer on broiler chickens’ microbiota, gut structure, and motility [17,18,19,20]. The use of Standardized Natural Citrus Extract (SNCE) is even more interesting as it helps to improve the carbon footprint of poultry farming, as demonstrated through the Life Cycle Assessment [21]. Moreover, it involves the use of botanical products with a stable and standardized composition, focusing on the active compound responsible for its biological activity. We previously demonstrated the positive effect of a Standardized Natural Citrus Extract (SNCE) on broiler chickens’ performance [18]. The question raised in that study was whether the SNCE’s positive effect on performance was correlated with an improvement in broiler chickens’ gut health, slaughter performance, and welfare. The hypothesis behind this study is that citrus extract would improve growth performance, meat quality, and litter quality compared to a standard situation, thanks to its positive effect on gut health. Broiler chickens’ welfare parameters linked to skin integrity would also be improved as a result.

## 2. Material and Methods

### 2.1. Ethics

All animal procedures were approved by the Warsaw University of Life Science (SGGW) 2nd Local Ethics Committee for animal experiments to ensure compliance with Polish and European regulations for animal welfare concerning the use of live vertebrate animals in research and teaching (waivers from 06.04.2023 of Ethics Committee approval).

### 2.2. Animals and Diet

Seven hundred fifty-six 1-day-old males (Ross 308, Aviagen, Zebowo, Poland) were randomly assigned to 2 groups: a control group (CTL) in which broiler chickens were fed with a standard diet whose characteristics fit with the Ross 308 specification feed, and a citrus group in which broiler chickens were fed with the same standard diet and supplemented with 250 g/T of Standardized Natural Citrus Extract (SNCE, Nor-Spice AB^®^, Nor-Feed SAS, Beaucouzé, France), with 14 and 13 replicates of 28 birds each, respectively. The complete characterization of SNCE has already been performed [19]. Each pen (1.45 m × 1.45 m) was equipped with a tube feeder and 2 bell drinkers during the first week of life. After that, bell drinkers were replaced by automatic nipple drinkers. Straw pellet was used as litter and birds had *ad libitum* access to feed and water throughout the trial. Chickens were reared until d35, starting at 34 °C at chick placement, gradually reduced to 25.5 °C by 25 days of age, where it was maintained from thereafter. Animals were exposed to artificial light according to Ross 308 broiler light program instructions. The chickens have had 23 h of light and 1 h of darkness from d0 to d7 and 5 h of darkness since day 7.

The feeding program was set up as follows: starter from d 1 to d 10, grower from d 11 to d 27, and finisher from d 28 until d 35. Nutritional values of the diets are described in Table 1. Coccidiostat was not used in the feed.

### 2.3. Growth Parameters and Slaughter Performance Assessment

Body weight per bird was recorded weekly from d 1 to d 35, whereas feed intake (FI) was measured in each dietary transition (d 10, d 27, and d 35). Feed conversion ratio (FCR) was also calculated at d 10, d 27, and d 35, and mortality was recorded daily. Based on the parameters monitored, the European Efficiency Index (EEI) was calculated using the following formula:

$$EEI=\frac{average\;body\;weight\;(kg)\;\times \;survival\;rate\;(\%)}{rearing\;days\;\times \;feed\;consumption\;per\;kg\;body\;weight}$$

Regarding slaughter performance, ten birds per group were randomly selected and weighed before slaughter. After slaughter and cooling (24 h in 4 °C) of the carcasses, the slaughter efficiency of the chickens was assessed based on the work of Michalczuk et al. 2016, determining the percentages of the pectoral muscle, leg muscle, giblets, and fat [22].

### 2.4. Gut Health

#### 2.4.1. Short-Chain Fatty Acids (SCFA) Analysis

Twenty birds per group were randomly selected among those within the group average weight of +/− 200 g for short-chain fatty acid (SCFA) analyses in the caecal digesta. The concentrations of SCFAs in caecal digesta were measured with the following modifications: 4 mL of ultrapure water was added to the caecal digesta, and the pH was determined using a WTW pH/340 pH meter (Fisher scientific, Illkirch, France). The SCFAs were converted to their respective sodium salts by adjusting the pH to 8.2 with 1 M NaOH. The samples were then stored at −20 °C. Before analysis, the samples were thawed at room temperature, thoroughly mixed, and centrifuged at 1000 rpm for 10 min at room temperature. The supernatants were collected, and formic acid was added to each sample in an amount equal to 10% of the sample volume. After mixing, the supernatants were centrifuged again at 10,000 rpm for 10 min at room temperature. A volume of 500 µL of supernatant was transferred into chromatographic vials and mixed with isocaproic acid (internal standard; IS) in a ratio of 15 µL of IS to 100 µL of supernatant. The samples were analyzed in duplicate using an HP 5890 Series II gas chromatograph (Hewlett-Packard, Waldbronn, Germany) with a flame ionization detector (FID) and a Supelco Nukol fused silica capillary column (30 m × 0.25 mm i.d.; 0.25 µm). Helium was used as the carrier gas with a flow rate of 103 mL/min. The oven temperature was initially set to 100 °C for 2 min, then ramped at 10 °C/min to 140 °C and held for 20 min. The injector temperature was maintained at 220 °C, and the detector temperature was set at 250 °C. The total analysis time was approximately 27 min. The concentrations of individual SCFAs were calculated relative to the internal standard using a mixture of SCFA standard solutions.

#### 2.4.2. Jejunal Permeability and Health Status

Jejunal permeability was assessed by measuring each group’s transepithelial electrical resistance (TEER) of 10 jejunum samples. Approximately 15 cm long jejunal segments distal to Meckel’s diverticulum were collected from each broiler after slaughter and rinsed with warm 0.9% NaCl solution. Tissues were then immersed in Krebs–Henseleit buffer solution (39 °C, pH 7.3) and transported to the laboratory. The ex vivo assessment of TEER was performed using the Single Channel Ussing chamber (Warner Instruments, Inc., Model U-9926, Hamden, CT, USA) with a jejunal tissue exposure area of 0.283 cm^2^. Jejunal segments for analysis were prepared according to the protocol described by Ruhnke (2013) with minor adjustments [23]. TEER was conducted using the Epithelial Voltage Clamp (Warner Instruments, Model EC-800, Hamden, CT, USA).

Briefly, samples were cut longitudinally on the mesenteric side, rinsed, and placed on a wooden board with the mucosal side facing upwards. Then, the tunica mucosa was scraped from the underlying tunica muscularis and tunica serosa in approximately 1.5 cm long strips. The collected mucosal explants were separated from the original tissue with scissors and then placed in Ussing chambers filled with Krebs–Henseleit buffer and continuously saturated with carbogen gas, maintaining a temperature of 39 °C and a pH of 7.3. Measurements were initiated after a 5 min equilibration period.

#### 2.4.3. Histopathological Examination

A veterinary histopathologist performed the evaluation in a blinded manner. Ten jejunal samples from 5 replicates of each group (Control vs. SNCE) were routinely fixed in 10% neutral buffered formalin, embedded in paraffin (Paraplast, Sigma-Aldrich, Saint Louis, MO, USA), and subsequently cut on a rotatory microtome. Preparations of 4 µm thickness were automatically stained with hematoxylin and eosin (HE, Varistain Gemini Thermo Scientific, Winsford, UK).

The microscopic analysis of HE slides was carried out with a BX43 light microscope equipped with an SC30 digital camera (Olympus Optical, Hachioji, Japan). Pictures were analyzed and recorded by computer software (CellSens Entry 2011V4.3.1, Olympus Lifescience, Tokyo, Japan). The presence of pathological changes was observed.

### 2.5. Welfare Assessment

Chicken welfare was evaluated according to the Welfare Quality Assessment Protocol for poultry (https://welfarequalitynetwork.net accessed on 13 November 2023). Parameters such as Footpad Dermatitis (FPD), litter quality, lameness (gate score), and hock burn have been assessed at d35, as described below. All the observations were performed by the veterinarian in charge of the flock in each broiler chicken from each pen in a blinded manner:

#### 2.5.1. Footpad Dermatitis

The assessment of FDP consisted of determining the severity of each broiler chicken lesion on a scale of 0, 1, 2, according to the table below (Table 2). The skin quality of the footpad was visually determined following the scale adopted by the Chief Veterinary Officer (pursuant to Art. 13 para. 1 pt. 1 of the Act of 29 January 2004 on Veterinary Inspection (Journal of Laws 2016, item 1077, as amended)).

#### 2.5.2. Litter Quality

Litter quality was evaluated according to the litter quality scoring scale described below (Table 3):

#### 2.5.3. Lameness (Gate Score)

Lameness assessment consisted of placing the birds on the floor within a long, straight section of the corridor and evaluating their ability to move according to the criteria below (Table 4). The evaluation was performed on each bird from the 2 groups:

#### 2.5.4. Hock Burn

**Figure 1:**
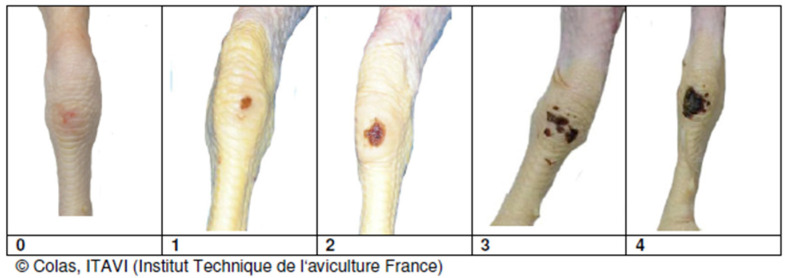
Hock burn visual assessment (http://www.welfarequalitynetwork.net accessed on 13 November 2023).

Hock burn is a contact dermatitis found on the skin of the caudal (back) part of the hock joint. The skin is turned dark by contact with litter, and skin lesions can result as a consequence. The scoring scale used for hock burn lesion severity assessment is presented in Table 5 and Figure 1 below:

### 2.6. Statistical Analysis

Statistical analyses of growth performance data and SCFA caecal concentration were performed by GraphPad Prism 7 software (GraphPad Software, San Diego, CA, USA). The pen was considered as a statistical unit for all growth performance parameters (*n* = 13 in the SNCE group and 14 in the CTL group). Statistical analyses were performed using Student’s test (*t*-test). The Shapiro–Wilk normality test was previously performed to determine if data were parametric, and the Grubbs test was performed to identify outliers. Regarding welfare analysis, statistical analyses were performed using the Mann–Whitney U test. Statistical significance was considered at *p* < 0.05.

## 3. Results

### 3.1. Growth Parameters and Slaughters Performance

The effects of SNCE supplementation on growth performance are presented in Table 6.

During this study, mortality was, respectively, 3.03% and 2.75% for the control and SNCE groups without any statistical difference between the two treatments (*p* = 0.761). SNCE supplementation improves broiler chickens’ body weight at d 35 (*p* < 0.001). The feed intake of broiler chickens was also different between the control and SNCE groups (*p* < 0.001). However, no difference was observed in the FCR of broiler chickens fed with SNCE compared to the CTL group (*p* = 0.340). The European Efficiency Index was also higher in the SNCE group compared to the CTL group (*p* < 0.001).

Results regarding slaughter performance are presented in Table 7 below:

Broiler chickens from the SNCE group had (*p* < 0.001, *t*-test) higher carcass weight compared to broiler chickens from the CTL group. The carcass yield tends to be higher in the supplemented birds compared to the CTL birds. Regarding organ weight, broiler chickens from the SNCE group had a (*p* = 0.009) higher proportion of breast muscle by 9.4% compared to broiler chickens from the CTL group. Additionally, the carcasses of SNCE chickens were characterized by a lower proportion (*p* = 0.025) of carcass fat, reduced by 20.6% relative to the CTL group.

### 3.2. Gut Health

SCFA concentrations in broiler chickens’ caecal digesta are presented in Table 8:

Results showed differences in terms of iso-butyric (*p* = 0.037) and iso-valeric acid (*p* = 0.003) concentrations in the caeca in favor of the CTL group, compared to the SNCE group. The putrefactive SCFA concentration was also lower in the SNCE group compared to the CTL group (*p* = 0.003).

**Figure 2:**
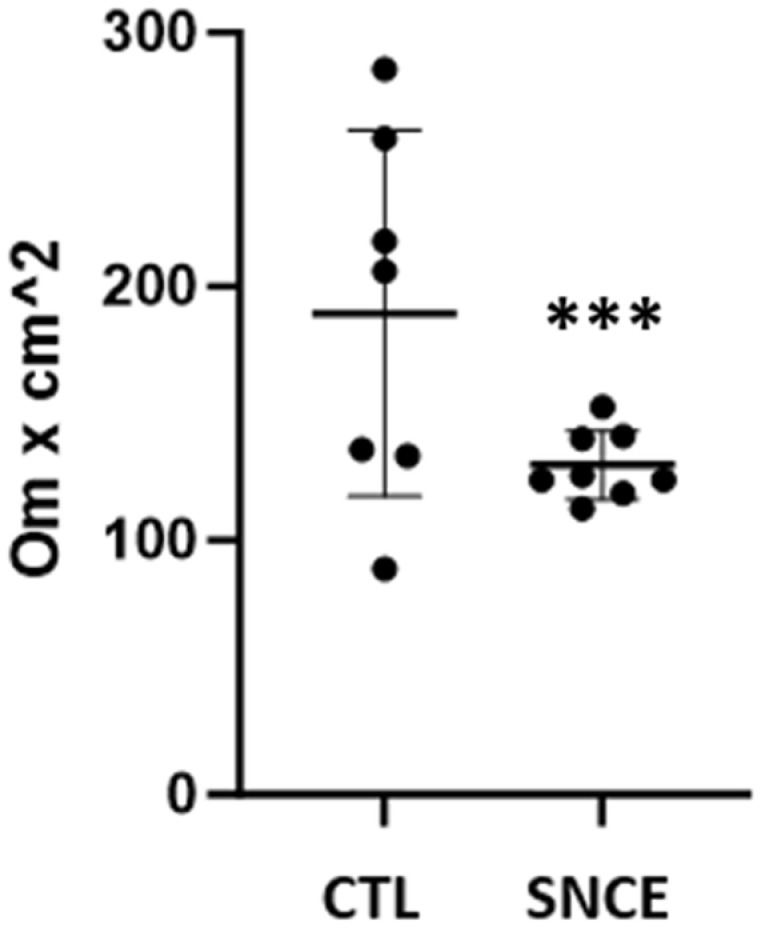
Transepithelial electrical resistance values from CTL and SNCE groups. *** *p* < 0.01.

Regarding the jejunal permeability evaluated through TEER analysis, results are presented in Figure 2.

The results showed that the TEER value in the control group was higher (*p* = 0.003) compared to the SNCE group.

The histopathological examination figures from one representative sample per group are presented in Table 9.

Histopathological observations showed that the jejunum samples from the SNCE group were characterized by scant inflammatory cellular infiltrates while the inflammation was severe in the CTL group (Table 9). Moreover, connective tissue hyperplasia was visible within the mucosa in the control group compared to the SNCE group. In addition, goblet cells from the SNCE group were strongly filled with mucus compared to goblet cells from the SNCE group. To finish, numerous mitotic figures were observed in samples from the SNCE group compared to the control group in which single or no mitotic figures were observed.

### 3.3. Welfare Assessment

The effect of SNCE on broiler chickens’ well-being is presented in Table 10:

Chickens’ welfare evaluation revealed significant differences in footpad dermatitis and gate score in favor of the SNCE group compared to the control group. Indeed, the percentage of chicken with FPD and gate score 0 was significantly higher in the SNCE group (*p* < 0.001, Mann–Whitney U test) compared to the control group. However, no significant difference was observed in litter quality. Regarding hock burn, chickens from the control group tended to have lower hock burn scores (*p* = 0.084, Mann–Whitney U test) than those from the SNCE group.

## 4. Discussion

### 4.1. Effect of SNCE on Growth Performance of Broilers Chickens

Regarding growth performance, findings from this study are in agreement with the results of our previous study, in which we reported that SNCE at 250 g per ton of feed positively influences the final body weight of broiler chickens [18]. However, contrary to the current study, no difference was observed in broiler chickens’ feed intake. The same trend was reported by Boumezrag et al. (2018), who observed an effect of SNCE on broiler chickens’ final weight [24]. Nevertheless, feed intake measurement was not evaluated in their study. Other studies also demonstrated a positive effect of SNCE on broiler chickens’ growth performance, i.e., final body weight and FCR [19,25]. Altogether, these results confirmed the interest of SNCE as a feed additive to improve broiler chickens’ growth performance. Conversely, many published studies performed with citrus products did not show a positive effect of their dietary supplementation on broiler chickens’ growth performance [17,26,27]. The lack of standardization of the used product may explain these inconsistencies in the observed effect. Indeed, citrus products used in animal nutrition can vary a lot in terms of composition but also the concentration of active compounds. This is mainly due to the part of the plant used, the used solvent in the case of extract, and to a minimum extent to the cultivar. Thus leading to a variation in the bioactive compound and consequently to various effects on performance [18]. SNCE standardization has already been demonstrated in our previous study [18] and may explain the replicable results over time.

### 4.2. Effect of SNCE on Slaughter Performance

Regarding slaughter performance, a positive effect of SNCE was observed on broiler chicken carcass weight and carcass yield. Similarly, Boumezrag et al. (2018) also demonstrated a positive impact of SNCE on carcass yield, showing an increase of 5.08% compared to the non-supplemented group. The close relationship between final body weight and carcass yield may explain these results [28]. Interestingly, SNCE supplementation also positively affects broiler chickens’ fat percentage and breast muscle weight. These results are consistent with Ebrahimi et al. (2014), who observed that *Citrus sinensis* peel extract dietary supplementation at 1250 g/T impacted the abdominal fat content of broiler chickens [29]. As reported by Tumovà et al., the ratio of dietary protein to energy is the factor with the greatest effect on fat deposition in broiler chicken [30]. Indeed, fat deposition happens when the energy balance is positive, resulting in excess energy needed for growth [30]. This suggests that SNCE dietary supplementation may have a positive effect on protein digestion and/or absorption and energy utilization to produce muscle, which may lead to less fat deposition. This assumption is reinforced by the fact that carcass weight and yield, as well as the breast muscle weight, were also improved in the supplemented birds. Other authors highlight a mode of action related to the pH reduction in the intestine, which may interfere with the abundance of fatty acids [29], or microbiota modulation, which is well described to regulate lipid accumulation [29,31]. This hypothesis is also plausible as we have shown previously that SNCE dietary supplementation modulates gut microbiota [32]. More studies are needed to confirm this hypothesis.

### 4.3. Effect of SNCE on Gut Health

Gut health was assessed through different parameters. Primarily, SCFA concentration was evaluated in the caeca at the end of the supplementation phase. Many studies have demonstrated that SCFAs play a significant role in the regulation of intestinal health in poultry [33]. In this study, no different effects were found between the two groups concerning the concentration of major SCFA (i.e., acetic acid, propionic acid, butyric acid, valeric acid, and total SCFA). This result was not expected because major compounds from SNCE such as pectic oligosaccharides [34] and hesperidin [35,36] are well described to promote the production of SCFA. Moreover, data from our laboratory that have been presented at the 2024 European Poultry Conference have already shown an increase in the SCFA caecal concentration in broiler chickens fed with SNCE [37]. Some authors suggest that higher absorption may sometimes explain the reduction in SCFA concentrations in the gut [38]. Further studies are needed to better understand these results. Results also showed that SNCE supplementation reduces the amount of putrefactive SCFA (i.e., iso-valeric, iso-butyric, and total putrefactive SCFA) in the caeca. Putrefactive SCFA is a group of organic acids that are produced during the putrefaction or decomposition of proteins by certain bacteria. The increase in their amount in the gut generally indicates adverse conditions, including a shift in pathogenic bacteria concentration in the intestine [39,40]. This result suggests that SNCE dietary supplementation in the gut may help improve protein absorption and/or modulate microbiota in favor of beneficial bacteria for the gut. It would have been interesting to perform digestibility assay or evaluate broiler chickens’ microbiota composition from this study to confirm this hypothesis. Nevertheless, these results are in line with the previous statement that SNCE could improve protein digestion and/or absorption.

Secondly, the integrity and permeability of the jejunum were assessed through TEER analysis. The results showed that TEER values from the CTL group were higher than TEER values from the SNCE group. However, TEER values from the CTL group were very heterogeneous, with some individuals with very high TEER values (over 200 Ω·cm^2^), which may be a sign of an inflammatory process, tissue scarification, or excessive mucus production. This is in accordance with the results presented by Yuan et al. (2020), where the increase in TEER was associated with mild cell layer damage. According to Srinivasan et al., 2015, TEER values for small intestine models using a Ussing chamber should be between 50 and 100 Ω·cm^2^ [41]. In their study, the increase in TEER alone was explained as a result of bacterial adhesion, ion transporters dysfunction, and mild structural damage to the cell layer [42]. Moreover, TEER values from SNCE were more uniform, which may indicate a similar health status of the tested animals. The variation between individual animals was greater in the CTL group, suggesting differences in intestinal health between broiler chickens.

Thirdly, histopathological observations were performed on jejunal samples. These findings are in accordance with our previous results on TEER, confirming that the increase in TEER value in the CTL group was correlated with an inflammatory process in the gut. It is well known that multiple environmental factors, such as changes in feed formulation or high-energy diets, can initiate gut inflammation [43,44]. The reducing effect observed from SNCE may be explained by SNCE composition. Indeed, it has been demonstrated in our previous study that SNCE is mainly composed of pectic oligosaccharides and citroflavonoids [18]. Some of the compounds identified are well described for their beneficial effect on different gut compartments, including anti-inflammatory properties. For instance, Parhiz et al. (2015) showed that hesperidin treatment allows for a decrease in the prevalence of inflammatory mediators, including COX-2, iNOS, and NF-κB [45,46]. Eriocitrin and citric acids [47] are also well described for their anti-inflammatory properties. Altogether, these results support the concept that SNCE enhances intestinal health by improving nutrient absorption and gut integrity, which results in an increase in broiler chickens’ growth performance.

### 4.4. Effect of SNCE on Welfare

Results from this study showed that citrus extract dietary supplementation positively affects the occurrence of FPD and the gate score compared to the control group, indicating an improvement in broiler chickens’ welfare. These results are not surprising. Indeed, litter moisture is the most important factor associated with the prevalence of FPD [48]. It is well known that improving gut integrity may improve water retention in the gut, resulting in less wet litter and lower pododermatitis occurrence. However, surprisingly, no difference was observed in litter quality between the groups. It would have been interesting to measure other litter parameters, such as ammonia concentration or litter pH, that could also play a role in FPD prevalence and severity [49]. Moreover, a higher occurrence of FPD is associated with a high incidence of other types of contact dermatitis such as hock burns and breast blisters in most cases [48,50]. The contrary was observed in our study. Indeed, hock burn prevalence was increased in the SNCE group compared to the CTL group. Further studies are needed to explain these results better. One of the current challenges of broiler chicken production consists of maintaining good productivity and taking into account the societal demands regarding the production systems [8]. These societal demands include but are not limited to sustainability and animal welfare concerns. The obtained results showed that using SNCE as a feed additive has a positive impact on animal welfare. A previous study has already shown interest in using SNCE to mitigate the environmental footprint of the livestock production sector [21]. Altogether, our results suggest that SNCE is a promising solution to reconcile livestock productivity and sustainability while taking into account animal welfare.

## 5. Conclusions

Results from this study showed that the use of Standardized Natural Citrus Extract as a feed additive could be a good solution to improve broiler chickens’ productivity as well as gut health and welfare. Indeed, growth performance and slaughter performance were improved using SNCE. Meanwhile, SNCE markedly decreased the concentration of putrefactive SCFA in the gut, but it improved gut integrity. The occurrence of FPD and broiler chickens’ lameness was also decreased after SNCE dietary supplementation. Taken together, these results suggest that SNCE may act as an efficient feed additive to improve the productivity of broiler chicken production while taking into account the societal demands regarding animal welfare concerns.

## Figures and Tables

**Table 1:** Feed composition and nutritional value of standard diet used.

Components	Starter (1–10 d) (kg)	Grower (11–27 d) (kg)	Finisher (28–35 d) (kg)
Corn 8.2%	20	20	20
Wheat 11.5%	41.741	42.513	35.751
Triticale 10.5%	-	-	10
Soybean meal 46%	28.159	25.369	21.554
Rapeseed meal 35%	4	5	5
Soybean oil	2.315	4.013	4.773
L-lysine 78%	0.419	0.36	0.347
DL-methionine 98%	0.311	0.196	0.218
L-threonine 98%	0.141	0.091	0.108
Limestone	1.47	1.133	1.121
Mono-calcium phosphate	0.843	0.723	0.522
Salt	0.324	0.326	0.329
Vitamin–mineral premixture ^1^	0.25	0.25	0.25
Xylanase (Ronozyme WX, DSM, La Garenne-Colombes, France)	0.012	0.012	0.012
Phytase (Axtra ^®^ PHY GOLD, IFF-Danisco Animal Nutrition and Health, Brabrand, Denmark)	0.015	0.015	0.015
Total (kg)	100	100	100
ME (kcal/kg)	2950	3100	3150
Neutral detergent fiber (%)	3.43	3.43	3.31
Crude protein (%)	21.5	20.5	19
Total lysine (g/kg)	13.5	12.5	11.5
Total methionine (g/kg)	6.21	5	5.02
Met. + Cys. (g/kg)	10	8.7	8.5
Total threonine (g/kg)	9	8.2	7.8
Total tryptophan (g/kg)	2.55	2.43	2.23
Total arginine (g/kg)	13.35	12.68	11.58
Calcium (g/kg)	10	8.5	8
Phosphorus (g/kg)	6.87	6.55	5.89
Avail. phosphorus (g/kg)	4.8	4.5	4
Sodium (g/kg)	1.5	1.5	1.5
Chloride (g/kg)	3.3	3.18	3.11
	^1^ Provided per kilogram of diet: Vitamin A (E 672): 10.000 IU; Vitamin D3 (E 671): 4.000 IU; Vitamin E (a-tocopherol): 15.0 mg; Vitamin K3: 3.0 mg; Vitamin B1: 2.0 mg; Vitamin B2: 5.0 mg; Vitamin B6: 4.0 mg; Vitamin B12: 11.0 µg; Nicotinic acid: 40.0 mg; Calcium pantothenate: 12.0 mg; Folic acid: 2.0 mg; Biotin: 0.18 mg; Cu: 8.0 mg; Fe: 50.0 mg; I: 2 mg; Mn: 70.0 mg; Se: 0.15 mg; Zn: 80.0 mg.	^1^ Provided per kilogram of diet: Vitamin A (E 672): 10.000 IU; Vitamin D3 (E 671): 4.000 IU; Vitamin E (a-tocopherol): 15.0 mg; Vitamin K3: 3.0 mg; Vitamin B1: 2.0 mg; Vitamin B2: 5.0 mg; Vitamin B6: 4.0 mg; Vitamin B12: 11.0 µg; Nicotinic acid: 40.0 mg; Calcium pantothenate: 12.0 mg; Folic acid: 2.0 mg; Biotin: 0.18 mg; Cu: 8.0 mg; Fe: 50.0 mg; I: 2 mg; Mn: 70.0 mg; Se: 0.15 mg; Zn: 80.0 mg.	^1^ Provided per kilogram of diet: Vitamin A (E 672): 10.000 IU; Vitamin D3 (E 671): 4.000 IU; Vitamin E (a-tocopherol): 10.0 mg; Vitamin K3: 3.0 mg; Vitamin B1: 2.0 mg; Vitamin B2: 5.0 mg; Vitamin B6: 4.0 mg; Vitamin B12: 11.0 µg; Nicotinic acid: 40.0 mg; Calcium pantothenate: 12.0 mg; Folic acid: 2.0 mg; Biotin: 0.18 mg; Cu: 8.0 mg; Fe: 50.0 mg; I: 2 mg; Mn: 70.0 mg; Se: 0.15 mg; Zn: 80.0 mg.

**Table 2:** FDP measurement scale.

Score	0	1	2
Description	No evidence of FPD	Minimal evidence of FPD, superficial lesions, discolouration no more than 0.5 cm in diameter	Deep lesions with scab and ulceration, discoloration of 0.5 cm in diameter and larger
Picture	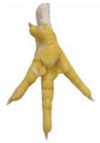	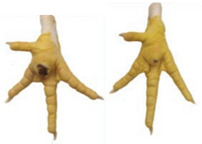	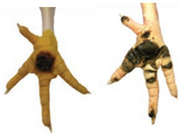

**Table 3:** Litter quality scoring scale.

Score	Description
0	Completely dry and flaky, i.e., moves easily with the foot
1	Dry but not easy to move with the foot
2	Leaves imprint of foot and will form a ball if compacted, but the ball does not stay together well
3	Sticks to boots and sticks readily in a ball if compacted
4	Sticks to boots once the cap or compacted crust is broken

**Table 4:** Gate scoring scale.

Score	Gate Description
0	Gate is smooth and the animal maintains balance while moving
1	Gate is irregular, unable to determine which leg is inoperative
2	Gate is irregular, the bird’s stride is shortened, and the chicken partially uses its wings for balance
3	Similarly to score 2, but the bird moves reluctantly and cannot stand for more than 15 s and lies down after a series of steps
4	The bird is reluctant to move and can only take a few steps in a series, the bird only keeps its balance with the constant help of its wings
5	Bird is unable to take a single step, even when forced to

**Table 5:** Hock burn scoring scale.

Score	Description
0	No evidence of hock burn (score ‘0’)
1	Minimal evidence of hock burn (score ‘1’ and ‘2’)
2	Evidence of hock burn (score ‘3’ and ‘4’)

**Table 6:** Dietary treatment effect on broiler chicken growth performance.

Parameters (d35)	Group				*p* Value
	CTL	SD	SNCE	SD	
Body weight [g]	2201.83	158.20	2364.34	43.93	0.001
FI/broiler [g]	3096.24	118.12	3281.44	84.57	<0.001
FCR [kg kg^−1^]	1.44	0.08	1.42	0.03	0.340
Mortality [%]	3.03	3.08	2.75	1.56	0.761
EEI [points]	426.31	24.61	461.24	13.67	<0.001

CTL: Control, EEI: European Efficiency Index, FCR: Feed Conversion Ratio, FI: Feed Intake, SD: standard deviation, SNCE: Standardized Natural Citrus Extract.

**Table 7:** Dietary treatment effect on broiler chicken slaughter performance.

Parameters	Groups				*p* Value
	CTL	SD	SNCE	SD	
Carcass weight [g]	1562.8	59.7	1639.6	75.9	<0.001
Carcass yield [%]	69.1	1.6	70.6	2.1	0.094
Breast muscles [g/100 g BW]	19.3	1.7	21.3	1.3	0.009
Drumstick [g/100 g BW]	13.0	0.9	12.6	1.9	0.526
Liver [g/100 g BW]	2.39	0.26	2.33	0.24	0.593
Gizzard [g/100 g BW]	0.69	0.07	0.67	0.14	0.727
Heart [g/100 g BW]	0.57	0.09	0.62	0.12	0.265
Fat [g/100 g BW]	1.36	0.28	1.08	0.24	0.025

Data are given as mean ± SD; CTL—control group; SNCE—experimental group.

**Table 8:** Concentration of short-chain fatty acids in the caecal digesta at 35 days of age.

Parameters	Group				*p* Value
[mM/g Caecal Digesta]	CTL	SD	SNCE	SD	
Acetic	3.92	0.78	4.17	1.00	0.382
Propionic	0.50	0.15	0.57	0.19	0.250
Iso-butyric	0.08	0.03	0.06	0.02	0.037
Butyric	0.94	0.29	0.88	0.27	0.467
Iso-valeric	0.11	0.04	0.07	0.02	0.003
Valeric	0.10	0.03	0.08	0.03	0.067
Total SCFA	5.62	1.09	5.86	1.39	0.547
Putrefactive SCFA	0.19	0.07	0.13	0.03	0.003

**Table 9:** Histopathological observation of jejunum from CTL and SNCE group.

	Observations	Picture 40×	Picture 400×
Samples from the CTL group	Inflammatory cellular infiltrates in the mucosa (with the participation of heterophils) Single mitotic figures in crypt epithelial cells Connective tissue hyperplasia	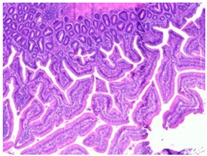	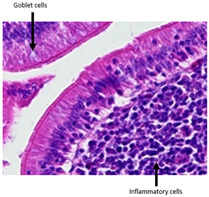
		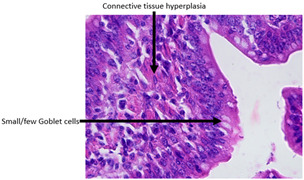	
SNCE group	Scant infiltration of inflammatory cells in the mucosa (with the participation of heterophils) and the formation of individual lymphatic nodules Numerous goblet cells/heavily filled with mucus Vesicular nuclei of intestinal crypt epithelial cells; numerous mitotic figures	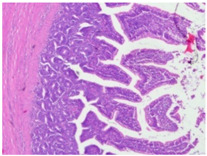	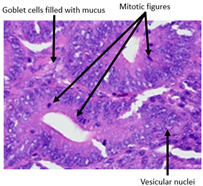
		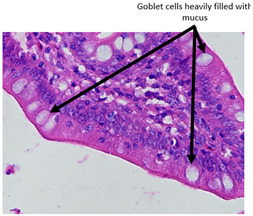	

**Table 10:** Assessment of chicken welfare at day 35.

Parameters	Score	Group				*p* Value
		CTL		SNCE		
		*n*	%	*n*	%	
Footpad Dermatitis	0	278	79.2	343	96.9	
	1	59	16.8	11	3.1	
	2	14	4.0	0	0.0	<0.001
Hock burn	0	329	93.7	317	89.6	
	1	22	6.3	37	10.4	0.084
Gate score (lameness)	0	298	84.9	343	96.9	
	1	53	15.1	11	3.1	<0.001
Litter quality	0	17	24.3	16	24.6	
	1	33	47.1	21	32.3	
	2	20	28.6	28	43.1	0.146

Statistical analyses were performed using the Mann–Whitney U test. Statistical significance was considered at *p* < 0.05.

## Data Availability

The data that support the findings of this study are available on request from the corresponding author, (S.C.).

## List of Abbreviation

C	Celsius
CTL	control
d	day
EEI	European Efficiency Index
FCR	feed conversion ration
FPD	Footpad Dermatitis
SCFA	Short-chain fatty acid
SNCE	Standardized Natural Citrus Extract
TEER	Transepithelial electrical resistance
